# An Ethical Analysis Regarding the COVID-19 Pandemic Impact on Oral Healthcare in Patients with Mental Disorders

**DOI:** 10.3390/healthcare11182585

**Published:** 2023-09-19

**Authors:** Oana-Maria Isailă, Eduard Drima, Sorin Hostiuc

**Affiliations:** 1 Department of Legal Medicine and Bioethics, Faculty of Dentistry, “Carol Davila” University of Medicine and Pharmacy, 020021 Bucharest, Romania; sorin.hostiuc@umfcd.ro; 2 Medical Clinical Department, Dunărea de Jos University, 800201 Galați, Romania

**Keywords:** oral health, COVID-19, mental disorders, justice, vulnerable, stigma

## Abstract

During the COVID-19 pandemic, restrictive measures were imposed that significantly impacted the healthcare system in general, and the dental healthcare system in particular. The literature cites a possible association between mental and oral health, as psychiatric patients have decreased awareness of their oral health and, therefore, poor dental status. Moreover, several studies have found a positive association between SARS-CoV-2 infection and oral health conditions, as well as between SARS-CoV-2 infection and mental health status. This context generated multiple ethical dilemmas in the case of persons with mental health disorders who require dental treatment because they are more vulnerable in this respect. This article aims to analyze the ethical issues in dental care for patients with mental disorders concerning the COVID-19 restrictive measures. The ethical aspects involved here are the basic principles of bioethics and the related elements of accessibility, equity, consent, and confidentiality.

## 1. Introduction

During the COVID-19 pandemic, restrictive social healthcare-related measures were imposed, which were required to properly manage a large number of infected patients, but also to protect citizens from disease transmission. The main route of transmission of the virus has been found to be airborne via small infected fluid particles (including aerosols) from the sick person when coughing, talking, sneezing, or breathing [1,2]. Thus, given the high risk of contamination with potentially dire consequences, many social, economic, or even medical services have been temporarily halted, leading to changes in living and working conditions. One of the medical areas that has been severely impacted by the pandemic, mainly due to its significant exposure to aerosols, has been outpatient dental care [3].

In this context, at the level of the general population, amid uncertainty and restrictive measures, common psychological reactions such as stress, anxiety, depression, frustration, panic, hopelessness, and despair, including self-harming behavior, were observed [4,5]. The impact of COVID-19 was disproportionate, with vulnerability being higher among persons with low socio-economic status and pre-existing somatic conditions [6]. A significant burden was identified in patients with pre-existing psychiatric conditions, both due to the overall pandemic context, which included lower accessibility to health services [6], higher risk of infection, and the SARS-CoV-2 infection itself, which has been shown to cause various short- and long-term mental health issues [7]. The literature cites a possible association between mental and oral health, as psychiatric patients have a decreased awareness of their oral health, a situation that was intensified by the limitations imposed by the pandemic context [8]. The interconnection between mental and oral health, following unhealthy behaviors and neglect of oral health, involves bacterial translocation and systemic inflammation against the backdrop of microbiome dysregulation, thereby observing a precarious dental status in conditions such as Alzheimer’s disease, depression, Parkinson’s disease, bipolar disorder, and Schizophrenia [9].

In the same manner, studies have found a positive association between SARS-CoV-2 infection and oral health conditions such as xerostomia, aphthous lesions [10], orofacial pain, and periodontal symptoms. Additionally, a positive association was observed between the severity of the infection and periodontal symptoms [11]. In the same sense, the specialized literature reveals possible positive associations between temporomandibular joint disease and the context of the SARS-CoV-2 pandemic, given the impact on the mental health of the population through the prism of isolation, social distancing, quarantine, and the infection itself. These increased the level of stress, anxiety, and depression, with stress being an aggravating factor for bruxism [12].

**Figure 1:**
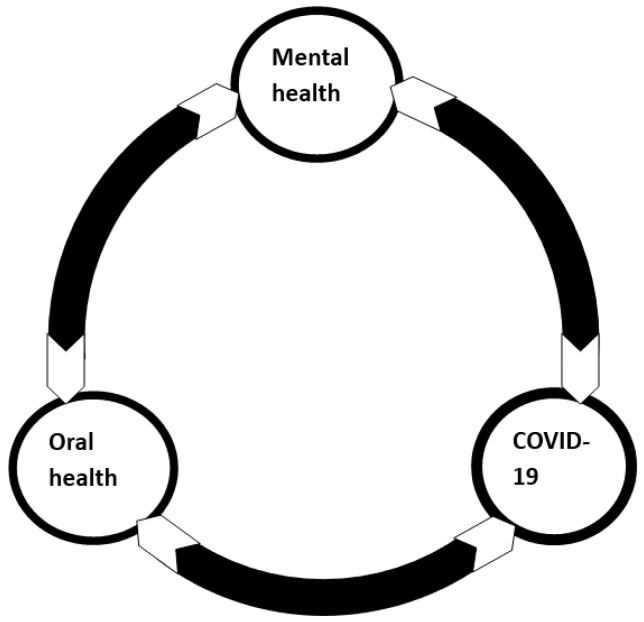
The interlinking association between COVID-19, mental health, and oral health.

Considering the above-stated facts, a complex interconnection between COVID-19, mental health, and oral health exists, which is summarized in Figure 1.

The main ethical issues in the dental care of patients with neurocognitive disorders before the COVID-19 pandemic, identified according to the literature on this topic, for example purposes, are listed in Table 1.

The purpose of our article is to analyze the ethical implications of the COVID-19 pandemic on oral healthcare in patients with mental illness.

## 2. Materials and Methods

We analyzed the scientific literature on moral principles and values related to oral healthcare for psychiatric patients during the COVID-19 pandemic. The search was conducted on Web of Science, Scopus, PubMed, and Google Scholar for peer-reviewed research that explores the dental approach to patients with psychiatric symptoms or disorders during the COVID-19 pandemic from a clinical and ethical perspective. The included literature focused on oral health structures in relation to the aforementioned populations with special needs, through the impact of the restrictive measures implemented during the pandemic, with their repercussions on the medical act and the patient. We also took into account scenarios centered on particular patient cases exposed to the general public, as well as the reactions and medical methods of adaptation generated.

## 3. Results

### 3.1. Beneficence and Nonmaleficence

As a general rule, during the early stages of the COVID-19 pandemic, dental beneficence has been deprioritized in relation to nonmaleficence, as the health-related risks associated with disease transmission, both for patients and physicians, have been considered higher than the consequences of postponing dental care [20]. Dental care has generally been limited to emergency cases during the pandemic to reduce the usage of scarce protective equipment [21]. Some authors have even recommended new frameworks to identify dental emergencies during the pandemic, based on a multilevel evaluation (oral, general, and psychological), associated with a risk assessment score to manage them [22]. Since the availability of protective equipment and vaccines, along with a better understanding of disease transmission and mechanisms, the deprioritization of beneficence has decreased. This has made oral care more accessible to patients regardless of their emergency status. Many patients with mental disorders who required dental consultation were institutionalized, lived in poorer communities, often lacked the material resources or protective equipment to travel to a dental practice or were unable to tolerate them, did not have proper access to newer technologies that were shown to facilitate dental assessment (such as telemedicine), were exposed to being infected as well as exposed their caregivers to being infected, were unable to respect general rules designed to minimize transmission, etc. [23,24,25,26]. Numerous measures have been implemented to prevent the transmission of infections and enable dental procedures. However, these measures were not always viable for individuals with mental health concerns. Dental organizations, for instance, devised protocols that called for conducting virtual consultations or telephonic conversations with patients before commencing treatment. This helped limit the number of physical interactions required [27]. Nonetheless, this course of action was not always a feasible option for patients with mental health conditions, as they either lacked the technical know-how or were disinterested in engaging with these modalities. The use of plexiglass shields, which were employed to separate the medical staff from patients to minimize direct aerosol exposure and limit personal contact, potentially increased patient anxiety and therefore decreased compliance [28]. These specific issues, associated with an overall reported increased difficulty in treating mental health patients by dental practitioners [29,30,31,32,33], have led to a longer deprioritization of dental beneficence in this population.

### 3.2. Veracity

Veracity is one of the pillars of the physician–patient relationship, involving therapeutic premises and patient expectations. Truthfulness confers respect for the autonomy of the patient or the parental/legal guardian authority in the decision-making process, with the patient’s best medical interests as its objective. To achieve the goal of veracity, physicians have a duty to prevent the promotion of misinformation, and therefore, speculative and misleading therapies. In the initial pandemic phase, external anxiety and caution prevailed in the absence of sufficient definitive evidence regarding COVID-19 to underpin medical practice in this regard [34]. In psychiatry, in the case of patients without decision-making capacity, one can speak of the physician–patient–legal representative relationship, a situation of increased complexity, when a person requires dental care against the background of the COVID-19 pandemic. Effective communication between physicians and patients is a pivotal component of medical care. However, several factors can impede this process. This may encompass challenges in accurately diagnosing and assessing the urgency of a patient’s condition when they are unable to articulate their discomfort. Additionally, prioritizing patients based on valid and ethical criteria, maintaining a solid physician–patient relationship while utilizing new equipment that limits nonverbal communication, and promoting patient adherence to treatment during physician–patient interactions can also pose significant obstacles [35]. Nonverbal communication is an important element in interpersonal relationships, even more so in a medical context, when patients with certain cognitive impairments are easily influenced by certain clothing and gestures. Trust and respect are largely inspired by nonverbal communication. For example, in the study by Lavelle et al., it was revealed that the nonverbal behavior of patients with schizophrenia can denote the avoidance of social interaction or the desire for social interaction, and this is associated with the behavior of their psychiatrist as well as the quality of the therapeutic relationship [36].

Furthermore, the COVID-19 pandemic has created uncertainty surrounding the accuracy of medical information as well as the effectiveness of certain treatments and investments in nonstandardized bio-decontamination equipment [37]. Studies in this regard have found varying levels of knowledge among the medical staff. For example, Wahed et al. found a knowledge level of 80.4% in respondents with higher education, and in those working directly with COVID-19-positive patients, a significantly increased knowledge score [38]. Similarly, Zhang et al. found that 89% of medical staff respondents had sufficient knowledge about COVID-19, and 89.7% followed correct practices regarding COVID-19 [39].

It is crucial for dental professionals to prioritize providing accurate medical information to patients [40]. However, the approach may need to be tailored to the patient’s physical and mental health conditions, social factors, and individual traits. Although truthful communication is typically expected by healthcare providers, conflicts may arise when certain values are at odds [41]. One such scenario that can be difficult for dentists is when a patient lacks the ability to make decisions, which may occur in those experiencing mental health disorders [42]. In such cases, uncertainty about the reliability of information may lead to anxiety for both the patient and the practitioner [43].

### 3.3. Consent and Confidentiality

Triage and informing patients of the risks of viral infection is mandatory, and confidentiality can be breached regarding the patient’s COVID-19 status with the maintenance of standards of care [44]. In particular, in patients with mental disorders, the possibility of absent/altered decisional capacity should be considered. For patients with impaired decision-making capacity, alternative, more suggestive methods of information can be used, with the minimization of medical terms, the use of beneficence centered physician–patient relationship models to decrease anxiety about making a complex decision, and the inclusion of the family in the decision-making process, with the explicit consent of the patient. Once the decision-making algorithm has been completed, the informed consent form is the sole responsibility of the patient, and the procedure is performed solely on the patient’s signature. In the case of patients whose decision-making capacity is absent, decisions are made by legal representatives, based on the patient’s previous wishes, concerning the patient’s current biological status and current dental needs [45]. The COVID-19 pandemic posed significant challenges to providing interventional dental treatment. Given the limited time for physician–patient interaction and the risk of contamination for the patient’s legal representative, the prioritization of dental emergencies was critical. Consent information also evolved due to the pandemic’s impact on acceptable procedures and available interventions. Drug treatment and tooth extraction emerged as the primary practices, while procedures involving aerosol use were avoided. Staged interventions that required constant monitoring were deferred until safe conditions were established [46]. Additionally, certain dentists have suggested implementing a written consent form to inform patients of the risk of COVID-19 transmission during dental appointments [47].

### 3.4. Justice, Accessibility, and Equity

The oral health of mental health patients and persons with special needs is frequently overlooked due to their lack of motivation and/or lack of awareness, dental treatment phobias, and poor economic situation. Common barriers such as decreased awareness, decreased expression of physical discomfort, and dependence on family members to reach the dental office or to provide treatment expenses are well known [48]. Pathological conditions, including oral health, can be assessed clinically, objectively, and strictly based on local examinations. The concept of “oral health-related quality of life” refers to the patient’s perception of the disease and its impact on their life. This is a multidimensional parameter that involves biopsychosocial elements. It can be influenced by cultural background, age, and other coexisting pathologies including mental disorders, with a major impact on the patient’s subjective perceptions [3,49]. In the context of the COVID-19 pandemic, these have been extended and augmented by restrictive measures, with reduced outpatient dental care and increased economic instability [50]. A scenario that reveals the amplification of the patient’s vulnerabilities is that of a person with paranoid schizophrenia with severe dental pain who has a panic attack when trying to visit the dentist’s office because is afraid of people with protective masks seen on the street [51]. If in the pre-pandemic period, the simple phobia of the dentist prevailed, during the pandemic, new phobias and new adjacent anxiety reasons emerged, making it more difficult for the patient to access treatment, in addition to the restrictive measures. Ensuring tailored accessibility for vulnerable populations with special needs is essential to avoid serious scenarios with irrevocable consequences and can also be a key factor in improving patient cooperation and compliance.

In the same sense, the absence of professional training for dentists regarding patient care for patients with special needs, the risk of stigmatizing this vulnerable population group, and the lack of training for psychiatrists on oral health screening [48], combined with the novelty of the insufficiently elucidated SARS-CoV-2 viral pathology, have increased barriers to dental care. Aljabri et al. identified anxiety, difficulty in keeping appointments, and treatment costs as the main barriers to accessing dental care among psychiatric patients [52]. In the context of the COVID-19 pandemic, Phadraig et al. found a decrease in the provision of dental care for people with disabilities during isolation, with the pandemic having an additional impact on their ability to access already scarce oral healthcare services, with dentists reporting significant decreases in pharmacological support for these patients [53]. Access to medical care was also further compromised in the case of people with dental emergencies who, due to neuropsychological impairments, were unable to express or become aware of their dental pathological status. Given the new working conditions, with the appropriate protective measures imposed, additional costs were incurred, which increased the cost of dental services [54]. A study conducted by Wolf et al. among dentists in Switzerland and Lichtenstein revealed that most dentists had to limit their dental practice to a minimum. By the end of 2020, 1.4% of dentists were forced to close their practice either permanently or temporarily due to a severely reduced economic situation [55]. Corollary to the aforementioned barriers, the pandemic has escalated these issues by increasing the incidence or worsening of mental disorders [7,56,57]. An obstacle frequently encountered by individuals with mental disorders seeking COVID-19 triage is the standardized protocol. To contain the spread of the virus, individuals were required to disclose whether they have been in contact with infected individuals or exhibit symptoms of the illness. However, this can pose a challenge to individuals with certain mental conditions. Knowledge and application of general and pandemic legislative frameworks, empathy, professionalism, and tact in treating these patients, with the establishment of psychiatrist–dentist collaborations were required [58], but at the same time, under the burden of the “duty to treat”, and the fear of contamination, physicians have experienced anxiety, which has led to reservations in approaching patients [59].

### 3.5. Autonomy and Phronesis: Patient versus Community?

Phronesis is based on practical experience and determines a person’s ability to perceive a situation to deliberate and act appropriately in given circumstances [60]. The pandemic has imposed caution and wisdom to minimize risk. Thus, public health measures took precedence over individual liberty to protect people with increased vulnerability and increased risk of death [61]. However, vulnerability is a hypothetical notion given the novelty of the etiological agent and insufficiently understood mechanisms of action. For example, in the study by Bain et al. among children with cystic fibrosis, SARS-CoV-2 infection was found to be clinically mild [62]. In pandemic management, moral resistance has played an important role in managing the ethical dilemmas generated in medical practice [63] by highlighting the physician as a public health agent at risk of compromising loyalty to the potential vector patient [64]. Delaying dental treatment in nonemergencies to control viral spread, according to some authors, has been associated with an “ethical barter for the greater good of the community” [46]. Patient autonomy and the need for regular dental care were undermined by an approach in which maximum caution dominated amid limited resources and incompletely understood infection mechanisms. This was more pronounced in people with mental disorders, in whom concern about oral health is much lower. According to a survey carried out by Knights et al. amidst the COVID-19 pandemic, dental health professionals expressed their primary concerns to be the potential of being accused of subpar treatment by patients, the sense of remorse for failing to provide adequate information to patients, postponed or missed diagnoses, and the disappointment of being unable to offer oral hygiene assistance to vulnerable patients with additional requirements [65].

For people with mental disorders, stigma can be extrinsic (public) but also intrinsic (self-stigma) [66]. In addition, SARS-CoV-2 infection has generated stigma and discrimination [67], given the exacerbation of negative attitudes toward already marginalized groups. This has created discussions about prioritizing the allocation of scarce resources, highlighting inequities in healthcare delivery among disadvantaged population groups [68].

The pandemic has caused a decrease in the number of dental procedures, leading to financial struggles in the dental profession. This is concerning, and there is a need for the profession to recover by prioritizing preventative services and ensuring equal access to oral health services [69].

### 3.6. Teledentistry and Its Implications

In the COVID-19 pandemic, telemedicine, which was already perceived as a promising tool for the prevention and promotion of oral health [70,71], gained momentum and became the preferred method given the background of protective measures. The main consideration behind this modern dentistry approach has been overcoming the limited personnel and infrastructure resources [72]. Teledentistry is considered reliable and feasible for patients with special needs for triage and treatment planning [73]. Remote oral examination via telecommunication is less stressful as it occurs in an environment familiar to the patient. At the same time, in the pandemic context, it has become preferable to avoid direct physician–patient interactions that could have favored the spread of the virus [74]. Rahman et al.’s research on the use of teledentistry among patients revealed that they were highly satisfied with the service. This study focused on factors such as the ease of use, increased accessibility to healthcare, reliability, and usefulness to patients. Over 90% of the respondents had a positive response to all parameters [75]. In their study among dentists on teledentistry, Cheuk et al. found that 49.3% of dentists use teledentistry, 36% since the pandemic. The three major uses of telestomatology by the respondents were in the areas of patient consultation and education. The mixed perception of this type of dentistry approach is derived from the lack of resources, lack of interest, and a limited dental curriculum to optimize its use [76]. In a study conducted among dentists and patients, Menhadji et al. revealed that 75.7% of the patients found the teleconsultation option more comfortable, and most of them showed a better understanding of their dental status, although they were initially skeptical about telemedicine. As far as dentists are concerned, those specializing in dental restorative processes considered telemedicine to be of little use in cases requiring specific in-person investigations for differential diagnosis. The barriers to teledentistry were technological because of the poor quality of service in the telecommunication network [77].

Although seemingly salutary given the particularity of the situation, teledentistry involves certain ethical challenges, including confidentiality, which may be compromised by breaches in information security [78] and informed consent, noting the risk of incomplete/inaccurate diagnosis given the remote physician–patient interaction. The validity of informed consent can be problematic in patients with mental disorders, as a person’s decision-making capacity is more difficult to assess via telecommunication [71,79].

### 3.7. Treatment Refusal vs. Duty to Treat

The duty to treat infected patients is based on four considerations: duty as an intrinsic moral obligation, duty as a response to a given trust, duty as a professional norm, and duty as an employer-imposed norm [45].

The provision of dental care involved medical risks for dentists in the initial pandemic phase with limited protective materials. Additionally, through the lens of neurocognitive disorders, a patient may be noncompliant or exhibit negative behaviors, which gives the clinician the right to refuse to initiate the therapeutic alliance as long as the patient’s dental status does not represent a dental emergency. Treatment refusal may be due to multiple causes, including cultural differences, inadequate (poor/excessive) information, information from different, contradictory sources (Internet, TV, magazines, relatives, etc.), physicians’ verbal and nonverbal communication, and medical conditions that may alter the decision-making process and implicitly lead to refusal of treatment [45]. According to Picciani et al., in the pandemic context, there has been an increase in dental emergencies in which uncooperative patients with neurocognitive deficits refuse dental care because of anxiety and fear, which would require dedicated dental sedation services as well as the behavioral management of anxiety and pain [80]. At the same time, this also implies an assessment of the patient’s decisional capacity to validate treatment refusal [45]. Pharmacological sedation techniques have been found to increase patient cooperation, create less traumatic therapeutic contexts, and allow for more personalized care. This approach is particularly beneficial for individuals with mild intellectual disability [81,82]. In the current pandemic situation, Alharbi et al. have categorized dental treatments into different groups. The first category is emergencies which include maxillofacial fractures, infections, and bleeding. The second category includes emergencies that can be treated with minimal invasion and without the production of aerosols, such as dental pain, dental fractures, and periodontitis. The third category includes emergencies that require invasive treatment and/or the use of aerosols. Nonemergencies such as asymptomatic pathologies fall into the fourth category. The fifth category includes elective treatment [20].

In the COVID-19 scenario, the refusal or interruption of the physician–patient relationship was questionable, with the physician having the obligation to treat in the situation of dental emergencies. In support of this obligation came the further obligation of the physician to have and use protective materials (mask, goggles, gloves, face shield, etc.) as well the patient’s obligation to provide the necessary information during the triage procedure about COVID-19 status, personal contacts, and symptoms. The drastic measures took into account the possible unfavorable consequences generated by the potential contamination of the physician, which would have required the interruption of his activity and the accentuation of the staff shortage, as well as the scenario of the physician becoming a vector of transmission of the SARS-CoV-2 virus both professionally (patients and colleagues) as well as on a social level [83], which, on the background of the epidemic, led to the premises for deprioritization in the provision of dental care for people with special needs, including patients with mental disorders with deficiencies in awareness of their dental status or even their pain and reluctance to seek specialist services. The survey conducted by Riguzzi and Gashi in June–July 2020 highlighted the marked concern of healthcare staff about patients, the elderly, and family members, and less about their own health. Increased levels of stress were detected owing to the lack of protective materials, staff shortages, and absence at the time of emergency structures and plans [84].

### 3.8. Future Perspectives

Although a higher prevalence of dental diseases among people with mental disorders is known, the issue of dental treatment is rarely addressed in the literature, which can be considered a limitation of this review. Suggestions to improve oral health in people with special needs include prophylactic dental programs in the context of interdisciplinary psychiatrist–dentist collaboration, the implementation of appropriate preventive dental measures, and easily accessible regular check-ups [85]. The implementation of protocols tailored to people with neurocognitive impairments that reduce anxiety and pain and the association of dental treatment with traumatic experiences is also required [80]. In light of the pandemic, there has been renewed focus on pre-existing challenges, and telemedicine has emerged as a reliable screening option for patients. Nonetheless, dentists must receive adequate training, and additional measures must be in place to uphold ethical standards and prioritize patients’ well-being [71]. As psychiatry and dentistry are two distinct medical fields, it would be beneficial to offer training for dentists to better understand the challenges that patients with neurocognitive disorders face when seeking and adhering to treatment.

## 4. Conclusions

The global impact of COVID-19 has rendered individuals with mental disorders more susceptible, and consequently, their dental healthcare has been adversely affected. It is crucial to acknowledge this reality and implement measures to prevent future circumstances that could inflict irreversible harm upon patients and the healthcare system. Safe and regulated means of obtaining dental services must be prioritized, while also analyzing the ethical implications of medical practices and their effects on vulnerable groups.

## Figures and Tables

**Table 1:** The main ethical issues in dental care for patients with neurocognitive disorders prior to the COVID-19 pandemic.

Authors, Year	Ethical Issues Identified
Nordenram et al., 1994 [13]	The treatment decision and treatment task
Nordenram and Norberg, 1998 [14]	The uncertainty regarding the provision of adequate medical treatment Respect for human integrity
Shuman 1999 [15]	Valid informed consent, ensuring patient safety and dignity
Gitto et al., 2001 [16]	Achieving the ethical norms without discrimination
Yao and MacEntee, 2014 [17]	Equity, accessibility
Delwel et al., 2017 [18]	The need to improve the training of health personnel for patient-centered dental treatment
Geddis-Regan et al., 2018 [19]	Medical personnel education for patient-centered dental treatment

## Data Availability

Not applicable.
